# The Frontal Assessment Battery in older multilingual Kenyan adults

**DOI:** 10.1002/alz70858_103492

**Published:** 2025-12-25

**Authors:** Anne Njoki Gitere, Anne Gitere, Anne Nyambura, Levi Muyela, Rachel Maina, Olivera Nesic‐Taylor, Jasmit Shah, Dilraj Sokhi, Sylvia Mbugua, Samuel Gitau, Sheila Ponda, Juzar Hooker, Zul Merali, Chinedu Udeh‐Momoh, Karen Blackmon

**Affiliations:** ^1^ Brain and Mind Institute, Aga Khan University, Nairobi, Kenya, Wake Forest University, Winston Salem, NC, USA, Dartmouth College, Hanover, NH, USA

## Abstract

**Background:**

The Frontal Assessment Battery (FAB) was developed as a short (6‐item) bedside assessment of executive dysfunction in older adults with suspected neurodegeneration. This study describes psychometric features of the FAB in multilingual Kenyan adults with and without clinical diagnosis of dementia.

**Method:**

The FAB consists of six items assessing verbal abstract reasoning, lexical fluency, motor learning, and inhibitory control. The score on each item ranges from 0‐3, with higher scores indicating better performance. Bilingual English‐Swahili study personnel translated, blind back‐translated, and applied consensus‐driven cultural adaptations. FAB was then administered to 95 participants [71 cognitively unimpaired (CU) controls; 24 people with dementia (PWD)] that ranged in age from 45 to 81 years (mean age = 59 years) and included 54 females and 41 males, with broad educational attainment (primary school to doctoral level). Psychometric evaluation included Cronbach's alpha to assess internal consistency and correlational analyses to assess age, sex, education, and language effects in the CU group. Group effects on FAB scores were assessed with multiple regression, after accounting for age, education, and language effects.

**Result:**

The 6‐item FAB shows good internal consistency (α = 0.85). Preferred language of assessment was the first language in 12%, second language in 55% and third language in 31%. There was no difference between first‐ and second‐language testing but a disadvantage was apparent in third‐language testing (mean diff = ‐1.3). No sex effects were apparent (*t* = ‐0.46; *p* = 0.65). FAB total scores were inversely associated with age (*rho* = ‐0.32; *p* < 0.01) and positively associated with education (*rho* = 0.4; *p* < 0.001). Group status predicted FAB scores (Beta = ‐0.72; *t* = 7.78; *p* <0.001), after accounting for age, education, and language effects. Average FAB scores for PWD were 9.3 (SD = 4.07) compared to 16.11 (SD = 2.1) in CU controls.

**Conclusion:**

Findings suggest sound psychometric features, with good internal consistency and expected age, education, and language effects. There were clear performance deficits in people with clinically diagnosed dementia, after accounting for important confounds, which suggest that the FAB may be a useful clinical tool for dementia detection in Kenyan adults.

